# Training medical students to conduct motivational interviewing: a randomized controlled trial

**DOI:** 10.1186/1940-0640-7-S1-A96

**Published:** 2012-10-09

**Authors:** Jean-Bernard Daeppen, Cristiana Fortini, Nicolas Bertholet, Raphael Bonvin, Alexandre Berney, Pierre-André Michaud, Carine Layat, Jacques Gaume

**Affiliations:** 1 Center for Alcohol Treatment, Department of Medicine and Public Health, Lausanne University, Lausanne, Switzerland; 2 Department of Biology and Medicine, University of Lausanne, Lausanne, Switzerland; 3 Department of Psychiatry, Lausanne University Hospital, Lausanne, Switzerland; 4 Department of Health Sciences, University of Lausanne, Lausanne, Switzerland

## 

Motivational interviewing (MI) is increasingly used to address unhealthy behaviors. We examined the effectiveness of MI training for medical students to improve counseling of patients with unhealthy behaviors, including risky alcohol use. All students (N = 131) in year five of a six-year curriculum at Lausanne University Medical School in Switzerland were randomized into an experimental group (n = 66) or control group (n = 65). After training of all students in basic communication skills in years two and three (control condition), an eight-hour MI training workshop was completed by students in the experimental group. One week after the training, students in both groups were invited to meet for 15 minutes with two standardized patients. Motivational-interviewing skills were coded by four blinded research assistants using the Motivational Interviewing Treatment Integrity (MITI) coding system, version 3.0. Superior performance was shown among trained versus control students as demonstrated by higher mean (standard deviation [SD]) scores (range, 1-5) for empathy (4.0 [0.6] versus 3.4 [0.7]; p < 0.001) and MI spirit (4.0 [0.6] versus 3.3 [0.6]; p < 0.001). Mean scores were similar between groups for direction, indicating that students in both groups invited the patient to talk about behavior change. Behavior-counts assessment demonstrated better performance in MI among trained versus control students regarding occurrences of MI-adherent behavior (mean [SD], 5.6 [2.5] versus 3.7 [1.7]; p < 0.001), MI nonadherent behavior (1.9 [2.3] versus 5.1 [3.7]; p < 0.001), closed questions (15.5 [5.3] versus 21.3 [6.9], p < 0.001), open questions (7.8 [2.9] versus 5.6 [2.1]; p = 0.001), simple reflections (13.2 [5.1] versus 11.1 [5.3], p = 0.03), and complex reflections (4.3 [2.1] versus 2.7 [2.0]; p < 0.001). Occurrences were similar between groups regarding giving information. In sum, an eight-hour training workshop was associated with improved MI performance, lending support for the implementation of MI training in medical schools.

